# Selected Serious Adverse Events in a Cohort of Adult ICU Patients: Protocol for a Sub‐Study of the PATIENCE Cohort

**DOI:** 10.1111/aas.70125

**Published:** 2025-09-18

**Authors:** Vera Crone, Morten Hylander Møller, Anders Granholm, Anders Perner, Waleed Alhazzani, Abdulrahman Al‐Fares, Johanna Hästbacka, Marlies Ostermann, Carmen A. Pfortmueller, Ricard Ferrer, Annika Reintam Blaser, Eric Keus, Wojciech Szczeklik, Mette Krag

**Affiliations:** ^1^ Department of Intensive Care Holbæk Hospital Holbæk Denmark; ^2^ Department of Intensive Care Copenhagen University Hospital – Rigshospitalet Copenhagen Denmark; ^3^ Department of Clinical Medicine University of Copenhagen Copenhagen Denmark; ^4^ Section of Biostatistics, Department of Public Health University of Copenhagen Copenhagen Denmark; ^5^ Scientific Research Centre Ministry of Defence Health Services Riyadh Saudi Arabia; ^6^ Department of Critical Care Medicine King Saud University Riyadh Saudi Arabia; ^7^ Department of Anaesthesia, Critical Care Medicine and Pain Medicine Al‐Amiri Hospital, Ministry of Health Kuwait City Kuwait; ^8^ Department of Intensive Care Tampere University Hospital, Wellbeing Services County of Pirkanmaa and Tampere University Tampere Finland; ^9^ Department of Critical Care King's College London, Guy's and St. Thomas' Hospital London UK; ^10^ Department of Intensive Care Inselspital, Bern University Hospital and University of Bern Bern Switzerland; ^11^ Vall D'hebron Hospital Universitari Vall D'hebron Barcelona Hospital Campus Barcelona Spain; ^12^ Department of Anaesthesiology and Intensive Care University of Tartu Tartu Estonia; ^13^ Department of Intensive Care Medicine Lucerne Cantonal Hospital Lucerne Switzerland; ^14^ Department of Critical Care University Medical Center Groningen Groningen the Netherlands; ^15^ Center for Intensive Care and Perioperative Medicine Jagiellonian University Medical College Krakow Poland; ^16^ Department of Anaesthesiology, Surgery and Trauma Centre Copenhagen University Hospital ‐ Rigshospitalet Copenhagen Denmark

## Abstract

**Background:**

Serious adverse events (SAEs) are a relevant patient safety concern in critically ill patients, yet epidemiological data on their occurrence in the intensive care unit (ICU) remain limited. This sub‐study of the PATIENCE cohort study aims to describe the occurrence of selected SAEs in adult ICU patients with and without prokinetic treatment.

**Methods:**

This is a protocol and statistical analysis plan for a post hoc sub‐study of the “Prokinetic agents in adult intensive care unit patients—An international inception cohort study (PATIENCE)” which has included 1440 acutely admitted adult ICU patients from 11 countries.

The primary outcome is the proportion of patients experiencing selected SAEs, assessed according to exposure to prokinetic treatment. Secondary outcomes include the proportion of patients experiencing each specific SAE, their distribution by treatment duration, and timing in relation to ICU admission and prokinetic use. Furthermore, patient characteristics for those with and without the SAEs will be described. Data will be presented descriptively.

**Conclusion:**

This sub‐study will use data from the international PATIENCE cohort study to provide epidemiological data on the occurrence of SAEs potentially related to prokinetic use in adult ICU patients.

## 1 Background

Serious adverse events (SAEs) are defined as any untoward medical occurrence that results in death, is life threatening, prolongs hospitalisation or leads to significant disability. A serious adverse reaction (SAR) is an event which is suspected to be related to a specific intervention [1]. In the ICU, distinguishing SAEs from SARs is challenging, as many such events also occur as a consequence of critical illness [2].

Prokinetic agents are used in ICU patients with feeding intolerance to enhance gastrointestinal motility [3]. However, their use may be associated with undesirable adverse reactions. Erythromycin has been linked to gastrointestinal side effects [4], metoclopramide to neurological side effects [5], and both agents to cardiac arrhythmias, especially at higher doses or with prolonged use [3, 5, 6]. The overlap between these adverse effects and symptoms of critical illness complicates causal interpretation [7, 8, 9].

Yet, evidence on the safety of prokinetic agents in ICU patients remains limited [3, 10, 11].

We recently conducted an international, prospective cohort study, the PATIENCE study, evaluating the use of prokinetic agents and the occurrence of selected SAEs in adult ICU patients [12].

This sub‐study aims to provide detailed epidemiological data on the occurrence of these selected SAEs potentially related to prokinetic use in the PATIENCE cohort and to compare SAE patterns between patients treated with prokinetic agents and those who were not.

## 2 Objectives

1. The proportion of patients experiencing selected SAEs across the following subgroups: patients not receiving prokinetic agents or who experienced all SAEs prior to receiving prokinetic agents, patients treated with erythromycin prior to the occurrence of any SAE, patients treated with metoclopramide prior to the occurrence of any SAE, and patients treated with a combination of erythromycin and metoclopramide prior to the occurrence of any SAE.
2. The distribution of the SAEs in relation to the duration of prokinetic treatment.
3. The timing of the SAEs in relation to index ICU admission and initiation of prokinetic treatment.
4. Characteristics of patients with and without the SAEs.

## 3 Methods

### 3.1 Design and Setting

The objectives of this sub‐study of the PATIENCE cohort study were developed before the closure of the main database, while the protocol was written afterwards.

### 3.2 PATIENCE Study

The PATIENCE study was an international, prospective cohort study including 1440 patients from 56 ICUs across 11 countries. In participating ICUs, all acutely admitted adult patients were enrolled during a 14‐day inception period between August 2024 and March 2025, as selected by the site investigators. Patients with planned admission to the ICU and those previously enrolled in the study were excluded.

Data were collected daily during ICU stay until discharge, death, or up to a maximum of 90 days, with final follow‐up on day 90 [12].

The aim of the PATIENCE study was to investigate the proportion of patients receiving prokinetic agents and to explore potential associations between the use of prokinetic agents and patient‐important outcomes including 90‐day mortality, days alive and out of ICU, days alive and out of hospital, and days alive without life support [12].

The PATIENCE study and this sub‐study were approved by the Legal Department of Scientific Research in the Region of Zealand (EMN‐2024‐03449) and registered in the Danish Research Centre, Region of Zealand (p‐2024‐16,493). All national investigators obtained the necessary approvals or waivers, and informed consent was sought from patients when required by national law.

### 3.3 Definitions

#### 3.3.1 SAE/SAR

SAEs are defined by the severity of the outcome, such as death, life‐threatening conditions or prolonged hospitalisation, regardless of causality. In contrast, SARs imply a reasonable suspicion that the event is related to a treatment [13]. As causality cannot be determined within the framework of this observational study, we chose to report on selected SAEs to strive for an objective and consistent classification of events. The selected SAEs included in this study were chosen based on their clinical relevance and the known pharmacological effects of prokinetic agents [5, 6, 10].

### 3.4 Event Classification

SAEs will be classified according to their timing in relation to prokinetic therapy. SAEs occurring on the same day as, or after, prokinetic treatment initiation will be classified as occurring during prokinetic therapy. SAEs occurring prior to initiation will be classified as occurring before prokinetic therapy.

Other definitions will follow those used in the primary PATIENCE cohort. Definitions relevant to the present study are described in detail in Supporting Information S2.

### 3.5 Data and Variables

In the PATIENCE study, data were collected daily from medical records. Baseline data were collected at ICU admission and included demographics, comorbidities, the Simplified Mortality Score for the Intensive Care Unit (SMS‐ICU) [14], the source of admission, surgery prior to ICU admission, and data on treatment with prokinetic agents and parenteral nutrition.

Data on life support, selected SAEs, and treatment with prokinetic agents were collected daily during ICU stay. Vital status and data on additional hospital admissions were collected on day 90 [12].

Patient characteristics and outcomes will be described using variables from the PATIENCE database as detailed below (definitions are presented in the supplement). No additional variables will be required.

#### 3.5.1 Baseline Variables

Comorbidities (chronic pulmonary disease, ischemic heart disease or heart failure, chronic liver failure, chronic renal failure, diabetes type 1 or 2).

Life support including respiratory support (invasive or non‐invasive respiratory support), vasopressors/inotropes and renal replacement therapy.

#### 3.5.2 Daily Variables

New cardiac arrhythmias requiring treatment.

New extrapyramidal symptoms requiring treatment.

Diarrhoea requiring treatment.

Vomiting with clinically important aspiration.

Data on treatment with prokinetic agents (type, dose, frequency and duration).

#### 3.5.3 Outcomes

Primary Outcome

The proportion of patients experiencing one or more SAEs stratified by the following groups:

1. Patients not receiving prokinetic agents or who experienced all SAEs prior to receiving prokinetic agents
2. Patients receiving erythromycin prior to the occurrence of any SAE
3. Patients receiving metoclopramide prior to the occurrence of any SAE
4. Patients receiving a combination of erythromycin and metoclopramide prior to the occurrence of any SAE

Secondary Outcomes

1. The proportion of patients experiencing each of the following SAEs:
  ○ Cardiac arrest
  ○ Cardiac arrhythmias
  ○ Extrapyramidal symptoms
  ○ Vomiting with clinically important aspiration
  ○ Diarrhoea > 1000 mL/day

reported within the same groups as defined for the primary outcome.

2 The proportion of patients experiencing any SAE after initiation of prokinetic therapy, stratified by longest consecutive treatment duration (≤ 3 vs. > 3 ICU days).
3 Time from index ICU admission to the first SAE.
4 Time from initiation of prokinetic treatment to the first SAE during index admission.

### 3.6 Statistical Considerations

#### 3.6.1 Statistical Considerations for Primary Outcome

Patients will be grouped by their first prokinetic treatment: single agent or combination. Group assignment will remain unchanged regardless of subsequent treatments. Given the anticipated low proportion of patients with single SAEs, all SAEs will be reported as a composite outcome. Patients will be included in the treatment group only if prokinetic therapy was initiated on the same day as, or before, any SAE. If all SAEs occurred prior to treatment initiation, the patient will be included in the non‐treatment group.

#### 3.6.2 Statistical Considerations for Secondary Outcomes

For the secondary outcome, each single SAE will be reported separately. If a patient experiences multiple SAEs, each event will be counted independently. As described above, patients will be grouped by their first administered prokinetic agent.

The remaining secondary outcomes will be presented descriptively using tables and figures, as appropriate.

Baseline characteristics will be described by SAE occurrence and prokinetic treatment, including age, comorbidities (0, 1, 2, 3+), use of life support (mechanical ventilation, vasopressor/inotropic therapy or renal replacement therapy) and reason for ICU admission.

### 3.7 General Statistical Considerations

#### 3.7.1 Sample Size

The sample size is fixed and determined by the PATIENCE cohort, which includes 1440 patients. Preliminary data suggest that ~355 patients (25%; 95% confidence interval (CI): 23%–28%) experienced one or more of the SAEs, with 14% having a SAE on the day of or after prokinetic treatment. These figures support the feasibility of the planned study.

### 3.8 Statistics and Reporting

We will present all data descriptively. We will summarise categorical data with numbers and percentages and continuous data with medians and interquartile ranges. Effect estimates will be reported with 95% CIs.

#### 3.8.1 Missing Data

We will report missing data as numbers and percentages. Some patients will be transferred directly from an ICU participating in the study to one that is not, where daily data were not collected. For these patients, we will assume that no events occurred on the days spent in the nonparticipating ICUs.

### 3.9 Publication Policy

We plan to submit the results from this sub‐study to an international peer‐reviewed journal. The manuscript will adhere to the Strengthening the Reporting of Observational Studies in Epidemiology (STROBE) statement [15]. Deviations from the protocol will be described. The protocol was prepared in accordance with the STROBE statement, and the checklist is provided in Supporting Information S1.

Authorship for the main manuscript of this sub‐study will be granted to members of the Management Committee and to national and local investigators according to the International Committee for Medical Journal Editors guidelines.

## 4 Discussion

The desirable effects of prokinetic agents in critically ill patients must be balanced against the undesirable effects. Both metoclopramide and erythromycin have been associated with cardiac arrhythmias, but the prevalence in critically ill patients remains unknown [16]. Most available data regarding SAEs related to prokinetic agents come from studies in non‐critically ill patients receiving erythromycin and metoclopramide at higher doses and for longer durations than typically used when prescribed as prokinetic agents in ICU settings [10]. Notably, no clinical trials included in a 2016 meta‐analysis of prokinetic agents in ICU patients had cardiac arrhythmias as a reported outcome [3].

This study will have some limitations. First, it is a post hoc sub‐study of the PATIENCE cohort, and the results should be considered exploratory. Second, data collection in the PATIENCE study was limited to the time spent in the participating ICUs, and potential events occurring outside these units were not included. In addition, the level of detail in this study will reflect the data available in the PATIENCE database. For instance, cardiac arrhythmias were recorded as any new arrhythmia, but we have no details on type or duration. Finally, we reported on SAEs, which, in contrast to SARs, are not suspected to be related to treatment, and no conclusions can be drawn regarding any relationship between prokinetic agent use and the occurrence of SAEs.

Strengths of this study include its large multinational ICU population, prospectively collected high‐quality data and the expected low proportions of missing data. As this sub‐study was pre‐specified prior to the closure of the primary PATIENCE database, the risk of selective reporting is reduced. Additionally, the use of standardised definitions for SAEs across sites is expected to enhance the reliability of the findings.

## 5 Conclusion

This sub‐study will provide international epidemiological data on SAEs potentially related to prokinetic use in a prospective cohort of adult ICU patients. By describing the occurrence and distribution of SAEs, these data will help inform the design of a future clinical trial on prokinetic agents.

## Author Contributions

Conceptualisation and study design: V.C., M.H.M., A.G., W.A., A.P., and M.K. Writing first draft: V.C., M.H.M., A.G. and M.K. Critical review and approval of manuscript: all authors.

## Conflicts of Interest

The Department of Intensive Care at Rigshospitalet—Copenhagen University Hospital (M.H.M., A.G., A.P.) has received funding from the Independent Research Fund Denmark, Novo Nordisk Foundation, and Sygeforsikringen “danmark” outside the submitted work. A.R.B. has received speaker or consultancy fees from Nutricia and VIPUN Medical and is holding a grant from the Estonian Research Council (PRG1255). J.H. has received reimbursement for advisory board work (Paion) outside the submitted work. Ricard Ferrer has received honoraria from Shionogi, MSD, Gilead, Menarini, Thermofisher, Viatris and AOP outside the submitted work.

## Supporting information


**Data S1:** Supporting Information.

## Data Availability

Data sharing not applicable to this article as no datasets were generated or analysed during the current study.
