# Supplementary material for: Selected Serious Adverse Events in a Cohort of Adult ICU Patients: Protocol for a Sub‐Study of the PATIENCE Cohort
Source: Acta Anaesthesiol Scand. 2025 Sep 18;69(9):e70125. doi: 10.1111/aas.70125 (PMC12445326; doi:10.1111/aas.70125)
Supplement: Supplementary file 1 — Data S1: Supporting Information. [file AAS-69-0-s001.docx]

**Supplementary material**

**Supplement to:**

**Selected serious adverse events in a cohort of adult ICU patients: Protocol for a sub-study of the PATIENCE cohort**

**Corresponding author:**

**Vera Crone, MD**

**veracrone@gmail.com**

**Table of contents**

[STROBE statement (S1) 2](#_Toc207008346)

[Definitions used in the PATIENCE cohort study (S2) 5](#_Toc207008347)

[References 9](#_Toc207008348)

### STROBE statement (S1)

STROBE Statement—checklist of items that should be included in reports of cohort studies

|  | Item No. | Recommendation | Page  No. |  |
| --- | --- | --- | --- | --- |
| **Title and abstract** | 1 | (*a*) Indicate the study’s design with a commonly used term in the title or the abstract | 1-3 |  |
|  |  | (*b*) Provide in the abstract an informative and balanced summary of what was done and what was found | 3 |  |
| Introduction | | | |  |
| Background/rationale | 2 | Explain the scientific background and rationale for the investigation being reported | 3-4 |  |
| Objectives | 3 | State specific objectives, including any prespecified hypotheses | 4 |  |
| Methods | | | |  |
| Study design | 4 | Present key elements of study design early in the paper | 4 |  |
| Setting | 5 | Describe the setting, locations, and relevant dates, including periods of recruitment, exposure, follow-up, and data collection | 4-5 |  |
| Participants | 6 | (*a*) Give the eligibility criteria, and the sources and methods of selection of participants. Describe methods of follow-up | 4-5 |  |
|  |  | (*b*) For matched studies, give matching criteria and number of exposed and unexposed |  |  |
| Variables | 7 | Clearly define all outcomes, exposures, predictors, potential confounders, and effect modifiers. Give diagnostic criteria, if applicable | 6-7 |  |
| Data sources/ measurement | 8* | For each variable of interest, give sources of data and details of methods of assessment (measurement).  Describe comparability of assessment methods if there is more than one group | 5-6 and supplement |  |
| Bias | 9 | Describe any efforts to address potential sources of bias | 8-9 |  |
| Study size | 10 | Explain how the study size was arrived at | 8 |  |

Continued on next page

| Quantitative variables | 11 | Explain how quantitative variables were handled in the analyses. If applicable, describe which groupings were chosen and why | 7-8 |  |
| --- | --- | --- | --- | --- |
| Statistical methods | 12 | (*a*) Describe all statistical methods, including those used to control for confounding | 7-8 |  |
|  |  | (*b*) Describe any methods used to examine subgroups and interactions |  |  |
|  |  | (*c*) Explain how missing data were addressed |  |  |
|  |  | (*d*) If applicable, explain how loss to follow-up was addressed |  |  |
|  |  | (*e*) Describe any sensitivity analyses |  |  |
| Results | | | | |
| Participants | 13* | (a) Report numbers of individuals at each stage of study—eg numbers potentially eligible, examined for eligibility, confirmed eligible, included in the study, completing follow-up, and analysed | NA |  |
|  |  | (b) Give reasons for non-participation at each stage |  |  |
|  |  | (c) Consider use of a flow diagram |  |  |
| Descriptive data | 14* | (a) Give characteristics of study participants (eg demographic, clinical, social) and information on exposures and potential confounders | NA |  |
|  |  | (b) Indicate number of participants with missing data for each variable of interest |  |  |
|  |  | (c) Summarise follow-up time (eg, average and total amount) |  |  |
| Outcome data | 15* | *Cohort study*—Report numbers of outcome events or summary measures over time | NA |  |
| Main results | 16 | (*a*) Give unadjusted estimates and, if applicable, confounder-adjusted estimates and their precision (eg, 95% confidence interval). Make clear which confounders were adjusted for and why they were included | NA |  |
|  |  | (*b*) Report category boundaries when continuous variables were categorized |  |  |
|  |  | (*c*) If relevant, consider translating estimates of relative risk into absolute risk for a meaningful time period |  |  |

Continued on next page

| Other analyses | 17 | Report other analyses done—eg analyses of subgroups and interactions, and sensitivity analyses | NA |  |
| --- | --- | --- | --- | --- |
| Discussion | | | | |
| Key results | 18 | Summarise key results with reference to study objectives | NA |  |
| Limitations | 19 | Discuss limitations of the study, taking into account sources of potential bias or imprecision. Discuss both direction and magnitude of any potential bias | 9-10 |  |
| Interpretation | 20 | Give a cautious overall interpretation of results considering objectives, limitations, multiplicity of analyses, results from similar studies, and other relevant evidence | NA |  |
| Generalisability | 21 | Discuss the generalisability (external validity) of the study results | 10 |  |
| Other information | |  | | |
| Funding | 22 | Give the source of funding and the role of the funders for the present study and, if applicable, for the original study on which the present article is based | 2 |  |

*Give information separately for cases and controls in case-control studies and, if applicable, for exposed and unexposed groups in cohort and cross-sectional studies.

**Note:** An Explanation and Elaboration article discusses each checklist item and gives methodological background and published examples of transparent reporting. The STROBE checklist is best used in conjunction with this article (freely available on the Web sites of PLoS Medicine at http://www.plosmedicine.org/, Annals of Internal Medicine at http://www.annals.org/, and Epidemiology at http://www.epidem.com/). Information on the STROBE Initiative is available at www.strobe-statement.org.

| **Variable** | **Points** |
| --- | --- |
| **Age** | |
| ≤ 39 years  40 – 59 years  60 – 79 years  ≥ 80 years | 0  5  10  13 |
| **Lowest systolic blood pressure** | |
| ≤ 49 mmHg  50 – 69 mmHg  70 – 89 mmHg  ≥ 90 mmHg | 6  5  3  0 |
| **Acute surgical admission** | |
| No  Yes | 3  0 |
| **Haematological malignancy  or metastatic cancer** | |
| No  Yes | 0  7 |
| **Vasopressors/inotropes ^a^** | |
| No  Yes | 0  4 |
| **Respiratory support ^b^** | |
| No  Yes | 0  5 |
| **Renal replacement therapy ^c^** | |
| No  Yes | 0  4 |
|  | |
| **Total score** | 0-42 ^d^ |

### Definitions used in the PATIENCE cohort study (S2)

**Simplified Mortality Score for the Intensive Care Unit (SMS-ICU)**

Reproduced from a previous paper^1^.

^a^ Continuous use of any vasopressor or inotrope.
^b^ Use of respiratory support, including invasive or non-invasive respiratory support and continuous use of continuous positive airway pressure (CPAP). Intermittent use of CPAP is not considered respiratory support. The PLOT-ICU study collected data on the use of mechanical ventilation only and did not consider non-invasive respiratory support and continuous CPAP.
^c^ Use of renal replacement therapy includes any renal replacement therapy whether chronic or acute, including continuous renal replacement therapy and intermittent haemodialysis, including the days in between intermittent haemodialysis.
^d^ Points assigned for the different variables in the score. It is not possible to obtain a total score of 1, 2 or 40 points. The worst value recorded during the first ICU is used^1,2^.

**Serious adverse events**

- Cardiac arrest

*Definition: The sudden cessation of cardiac activity*

- Cardiac arrhythmias requiring pharmacological treatment

*Definition: any cardiac arrhythmia requiring pharmacological treatment, including supraventricular tachycardia, ventricular tachycardia, ventricular fibrillation, or bradycardia*

- Extrapyramidal symptoms requiring pharmacological treatment

*Definition: Akathisia, dystonia, parkinsonism, or tardive dyskinesia* *requiring pharmacological treatment*

- Severe diarrhoea requiring treatment

*Definition: a volume of ≥ 1000 ml/day and/or if severe diarrhoea is noted in the medical record and requires treatment, e.g., fluids, stopping/pausing medication, or pharmacological therapy.*

- Vomiting with clinically significant aspiration requiring treatment

*Definition: entry of solid material into the trachea and lungs resulting in clinical deterioration with need for treatment (pharmacological or non-pharmacological) and/or aspiration requiring treatment is noted in the medical record.*

**Specification of baseline characteristics and comorbidities (relevant to this study)**

**Baseline patient characteristics**

**General patient data**

1. Main reason for ICU admission

- Neurological condition
- Respiratory failure
- Circulatory failure
- Renal failure
- Liver failure
- GI bleeding
- Other

**Comorbidities with definitions**

1. Chronic pulmonary disease

***Definition****: any history of chronic obstructive pulmonary disease, asthma, or other chronic lung disease or if the patient was treated at time of hospital admission with any relevant drug indicating chronic pulmonary disease, e.g. albuterol, levalbuterol, salmeterol, formoterol, arformoterol, indacaterol, vilanterol, olodaterol, tiotropium, aclidinium, umeclidinium, glycopyrronium, budesonide and fluticasone.*

1. History of severe heart failure (NYHA 3-4) or myocardial infarction

***Definition:*** *history of previous myocardial infarction, invasive intervention for coronary artery disease, stable or unstable angina, NYHA class 3 or 4 or measured LVEF < 40%.*

*NYHA class 3: marked limitation of physical activity. Comfortable at rest. Less than ordinary activity causes fatigue, palpitation, shortness of breath or chest pain.*

*NYHA class 4: symptoms of heart failure at rest. Any physical activity causes further discomfort.*

1. History of chronic liver failure

***Definition:*** *the presence of one or more of the following:*

- *portal hypertension*
- *cirrhosis proved by biopsy, computed tomography (CT) scan or ultrasound*
- *history of variceal bleeding*
- *hepatic encephalopathy in the past medical history*
- Ascites (non-malignant) in past medical history?

1. History of chronic renal failure

***Definition:*** *need for chronic renal support or S-creatinine > 3.6 g/dL / 300 μmol/L prior to hospital admission.*

1. Diabetes

***Definition:*** *treatment at the time of hospital admission with any relevant drug indicating diabetes, e.g., insulin, alpha-glucosidase inhibitors, biguanides (metformin), dipeptidyl peptidase-4 inhibitors (e.g., Januvia), Glucagon-like peptide-1 receptor agonists, meglitinides, sodium-glucose transporter 2 inhibitors, sulfonylureas, thiazolidinediones.*

**Daily data and definitions**

**Daily from ICU admission to the end of the 90^th^ day of follow-up, dead or discharge from ICU (whatever comes first).**

*(All values were collected daily while the patient was in the ICU. The timespan of the day form corresponds to calendar days, i.e., 00:00 – 23:59. The timespan of the day form will be shorter on days of admission, discharge, or death.)*

1. Continuous treatment with a vasopressor or inotropic agent on this day? (Y/N)

*The use of intermittent boluses is not considered a continuous infusion.*
*(E.g., epinephrine, norepinephrine, dobutamine, dopamine, phenylephrine, metaraminol, milrinone, levosimendan, angiotensin II, and vasopressin or one of its analogues)*

1. Treatment with continuous/intermittent renal replacement therapy on this day? (Y/N)

*Definition: any renal replacement therapy, whether chronic or acute, including continuous renal replacement therapy and intermittent haemodialysis, including up to three days in between intermittent haemodialysis*

1. Invasive mechanically ventilated on this day? (Y/N)
2. Was the patient treated with prokinetic agents on this day? (Y/N)

*Prokinetic agents: metoclopramide, erythromycin, domperidone, prucalopride*

1. Was any of the following specific serious adverse events observed on this day?

- Cardiac arrest
- Cardiac arrhythmias requiring pharmacological treatment
- Extrapyramidal symptoms requiring pharmacological treatment
- Severe diarrhoea requiring treatment
- Vomiting with clinically significant aspiration requiring treatment

**Extended day form for patients treated with prokinetic agents**

1. Dose and frequency of prescribed prokinetic agent

**Metoclopramide**:

*Dose*

*Frequency*

*Intravenously or orally*

**Erythromycin:**

*Dose*

*Frequency*

*Intravenously or orally*

**Domperidone:**

*Dose:*

*Frequency*

*Intravenously or orally*

**Prucalopride:**

*Dose*

*Frequency*

*Intravenously or orally*

**Specification of discharge data**

1. The patient is discharged to

- Ward
- ICU not participating in PATIENCE
- ICU participating in PATIENCE
- Home
- Death

1. Date and time for discharge (dd.mm.yyyy) and (hh:mm)
2. If death during the ICU stay (dd.mm.yyyy) and (hh:mm)

**Specification of follow-up data**

**Day 90 after the study index date**

- Vital status (death/alive)
- Death date (if relevant)

### References

1. Development and internal validation of the Simplified Mortality Score for the Intensive Care Unit (SMS‐ICU) - Granholm - 2018 - Acta Anaesthesiologica Scandinavica - Wiley Online Library. https://onlinelibrary.wiley.com/doi/10.1111/aas.13048.

2. Granholm, A. *et al.* External validation of the Simplified Mortality Score for the Intensive Care Unit (SMS-ICU). *Acta Anaesthesiologica Scandinavica* **63**, 1216–1224 (2019).
